# Circular RNAs Are Regulators of Diverse Animal Transcriptomes: One Health Perspective

**DOI:** 10.3389/fgene.2020.00999

**Published:** 2020-09-18

**Authors:** Dora Zucko, Kathleen Boris-Lawrie

**Affiliations:** Department of Veterinary and Biomedical Sciences, Veterinary Medicine Graduate Program, University of Minnesota Twin Cities, Saint Paul, MN, United States

**Keywords:** animal models, production animals, RNA biology, back-splicing, computational analysis, translational science, veterinary medicine, human health

## Abstract

Derived from linear (parental) precursor mRNA, circRNA are recycled exons and introns whose ends are ligated. By titrating microRNAs and RNA binding proteins, circRNA interconnect networks of competing endogenous RNAs. Without altering chromosomal DNA, circRNA regulates skeletal muscle development and proliferation, lactation, ovulation, brain development, and responses to infections and metabolic stress. This review integrates emerging knowledge of circRNA activity coming from genome-wide characterizations in many clades of animals. circRNA research addresses one of the main pillars of the One Health vision – to improve the health and productivity of food animals and generate translational knowledge in animal species.

## Historical Perspective

### Ubiquitous in Nature, Are RNA Circles Ancient Relics or Versatile Regulatory Molecules?

Linear polymers of DNA and RNA convey the flow of genetic information, whereas circularized forms of DNA and RNA serve regulatory roles in all domains of life. Given circularized DNA were identifiable before the advent of molecular biology and the current genomic-computational era, decades of study have established their significance.

The circularized DNA of mitochondria is necessary for energy generation, thereby governing growth, development and aging (Oldenburg and Bendich, 2015). Extrachromosomal circular DNA (EccDNA) are known to accumulate in the nuclei of healthy somatic cells (Møller et al., 2018). EccDNA structures encoding oncogenes accumulate in tumor cells and their abundant transcription drives oncogenesis (Von Hoff et al., 1992; Turner et al., 2017). Circular self-replicating plasmids and bacteriophages undergo autonomous rolling-circle replication and remodel host cell DNA through integration (Gilbert and Dressler, 1968; Dressler, 1970; Baas, 1985; Koonin and Ilyina, 1992; Ruiz-Masó et al., 2015; Wawrzyniak et al., 2017). DNA circles are significant in biological processes ranging from genome evolution to growth control and response to infection. This literature sets expectation circularized RNA (circRNA) are also highly significant.

CircRNA was first documented in viroids, infectious pathogens of higher plants (Sanger et al., 1976). The circular RNA structure was identified with biochemical evidence – viroid RNA was resistant to snake venom containing phosphodiesterase that degrades the phosphate on the 5′ end of linear molecules (Sanger et al., 1976). Evidence from phylogenetic studies posits viroid RNA replication is an ancient RNA relic of pre-cellular evolution (Diener, 1989). Viroids generate circRNA by complementary base pairing between RNA motifs on the same strand and in association with host RNA polymerase II (Zhong et al., 2008).

Similar to viroids, the circRNA of hepatitis delta virus (HDV) undergoes recombination and is assisted by hepatitis delta antigen. HDV is a defective human pathogen that is dependent on hepatitis B virus for replication in liver. Because of its circRNA genome, HDV has been considered unique amongst all known animal viruses (Taylor, 2014). In archaea, circRNA biogenesis involves remnants rRNA, tRNA intron and Box C/D RNAs forming ribonucleoprotein with dimeric RNA ligase (Rnl3) (Becker et al., 2019). The function of circRNA and primordial RNA binding proteins (RBPs) set the stage for eukaryotic circRNA biogenesis involving spliceosome RNPs (more below).

Recently, two gamma herpesviruses, Epstein-Barr virus (Ungerleider et al., 2018) and Kaposi’s sarcoma herpesvirus (Tagawa et al., 2018), were identified to generate circRNAs that are detectable in both the nucleus and cytoplasm of infected cells (Toptan et al., 2018). Levels of host circRNA decrease upon the viral infections, which may be due to reduced biogenesis, endonucleolytic decay by RNAse L (Li X. et al., 2017; Liu C. X. et al., 2019) or competition for host RBPs. Since host double-stranded (ds) RBPs are fundamental players in the host innate response to virus infection through binding of small viral RNA, virus-encoded circRNA may work to antagonize antiviral activity. Emerging evidence is viral circRNA prevent activation of protein kinase R, setting the stage to elucidate antagonism of antiviral response by exogenous circRNA (Chen et al., 2017; Liu C. X. et al., 2019).

A growing list of dsRBPs and splicing factors have been shown to mediate exon circularization and potentially may undergo sequestration and sorting by exogenous circRNA (for a recent review, see Huang et al., 2020). Future quantitative studies are speculated to reveal circRNAs are gene products of many animal viruses, which would raise the possibility for a novel diagnostic approach to detect these viruses. Significant primary sequence conservation has been discovered between circRNA of humans, old and new world monkeys, food animals, companion animals and murine species (Wang et al., 2014; Abdelmohsen et al., 2015; Chen et al., 2015; Ivanov et al., 2015; Venø et al., 2015; Zhang et al., 2016; Ouyang et al., 2018b; Qiu et al., 2018; Shangguan et al., 2018; Zhang F. et al., 2018). Sequence identity between circRNAs of mammals and flies indicate the fundamental steps in their biogenesis originated early in the evolution of life (Jeck et al., 2013; Memczak et al., 2013; Salzman et al., 2013). No doubt, circRNA are versatile regulatory molecules and their ubiquity in nature foretells important functions are yet-to-be discovered.

## Biogenesis and Function of Nascent Circular RNAs

### Making the Most of Precursor mRNA Sequences

**FIGURE 1:**
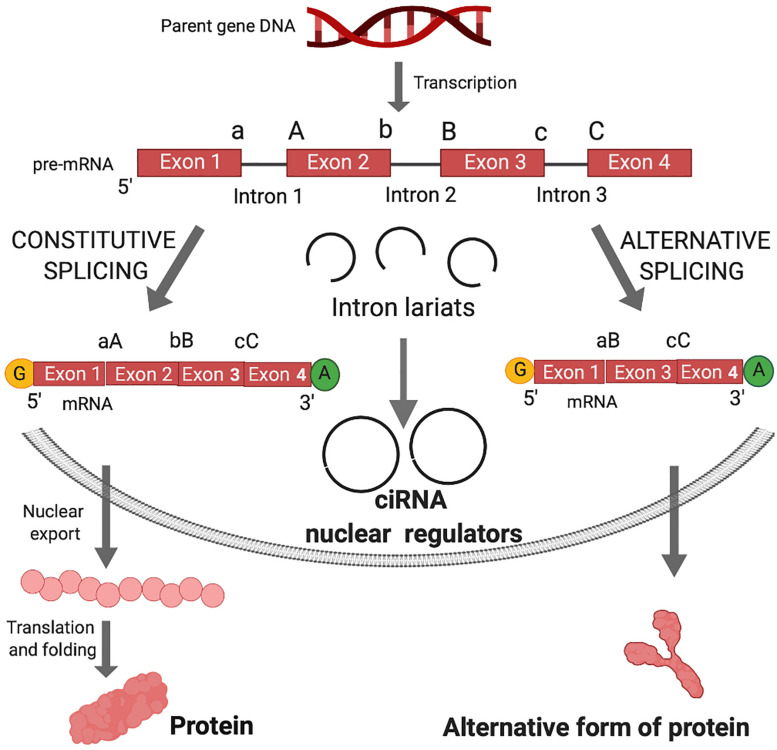
Canonical splicing and the biogenesis of circular intronic RNA (ciRNA) and functional outcomes. Shortly after gene transcription, the nascent RNA (pre-mRNA) is processed by the addition of the 7-methyl guanosine cap (yellow G) and polyadenylate tail (green A). In the linear splicing process, introns are excised and exons are joined in a linear manner to form mRNA for translation into proteins. Exons are joined by ligation of 3′ splice sites (a, b, or c) and 5′ splice sites (A, B, or C). The intron sequences are excised from the pre-mRNA creating intron lariats. Lariats can be destroyed in the process of debranching after excision from the pre-mRNA. In case of debranching failure, ligation of lariats can generate circular intronic RNA (ciRNA). ciRNA are retained in the nucleus and can act as nuclear regulators of parental gene transcription. Alternative splicing occurs when an exon in the pre-mRNA is skipped, resulting in diverse mRNA isoforms that may be either degraded via nonsense mediated decay or undergo nuclear transport, translation and folding into an alternative form of protein.

Eukaryotic circRNAs are generated during the co-transcriptional splicing of precursor mRNA (pre-mRNA) (Figure 1). Mature mRNA are decorated by 7-methyl guanosine (m7G) at the 5′ terminus and poly-adenosine residues at the 3′ terminus, modifications that are incompatible with covalent circularization of the linear RNA. The commitment to circRNA biogenesis involves poorly appreciated activity of spliceosome RNPs that is disengaged from canonical pre-mRNA processing. Functionally, the process has been characterized as mis-splicing or back-splicing (Jeck et al., 2013; Memczak et al., 2013; Yu and Kuo, 2019). The conserved patterns of back-splicing events reported in RNA from mouse, pig and human tissues supports the hypothesis that circRNA biogenesis is under positive selection (Venø et al., 2015; Yu and Kuo, 2019).

In canonical splicing of pre-mRNA, spliceosomes catalyze the excision of introns and ligation of consecutive exons (Figure 1). Alternative splicing engenders exon skipping to produce alternative open reading frames that encode unique protein isoforms (Figure 1). Spliceosomes activate 5′ splice site (ss) and 3′ ss for cleavage and ligation, while the intervening sequences between exons (introns) form lariat structures, which were long considered waste byproducts of linear splicing.

**FIGURE 2:**
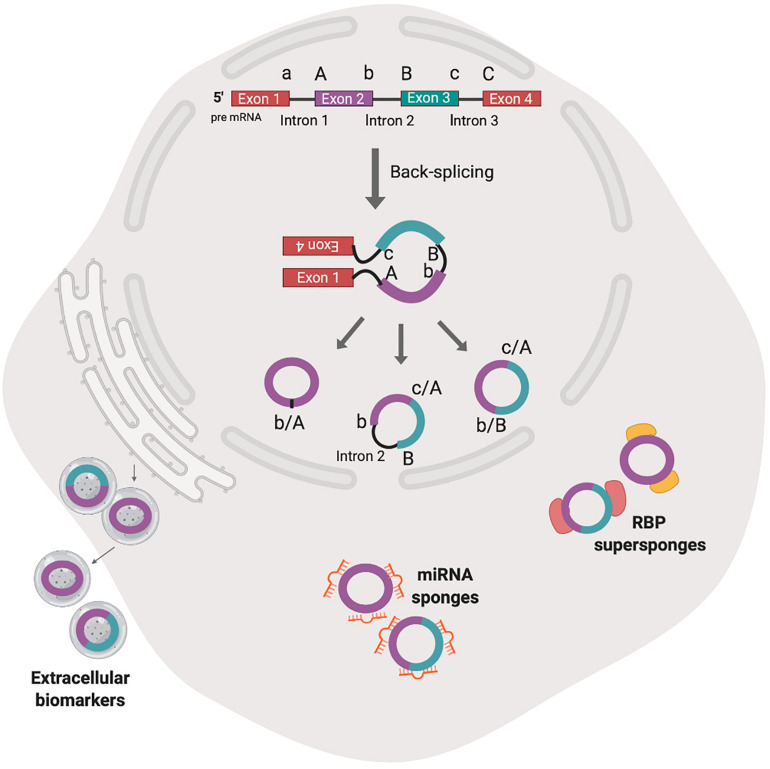
The biogenesis by non-canonical splicing and activities of animal circRNAs. Back-splicing model shown here is hypothesized to be the predominant mechanism of animal circRNA biogenesis. The non-linear combination of 3′ splice donor (a, b) and proximal 5′ splice acceptor (A, B) generates single exon circRNA (b/A), backspliced exons (c/A, b/B) or exons and introns (c/A, b intron 2 B). After nuclear export, the circRNA have been shown to accumulate in exosomes and may serve as extracellular biomarkers. Cytosolic circRNAs have been shown to interact with cognate microRNA (miR) and RNA binding proteins (RBPs) (sponge) in competition with miR response element on target mRNAs or another protein partner.

Non-canonical splicing has been characterized by delayed or aberrant spliceosome activity (Dvinge, 2018). Ligation of a 5′ ss with an upstream 3′ ss generates a covalently closed RNA molecule (Figure 2). The advanced computational analysis of RNAseq data has elucidated joints between 3′ and 5′ ss of non-consecutive exons and the joining of intron sequences at the branchpoint on a genome-wide scale (described in the next section). Computational analysis together with specialized protocols to enrich circRNAs in RNAseq samples, has fueled the explosion of circRNA research in the past 5 years. NCBI reported ∼5,000 new publications in the term 2015–2020 compared with 5,000 in the prior 50 years (1964–2014) (Pubmed July 20, 2020). As described in following sections, circRNA activity significantly expands the functional capacity of pre-mRNA, just as the protein coding capacity is amplified by alternative splicing.

### A Prelude to Discovering a Full Repertoire of Functionalities: Nuclear Interactions Regulate the Fate of Natural circRNA

Although a majority of circRNA derive from protein-coding RNAs, these molecules are considered long non-coding RNAs (lncRNAs) (Yu and Kuo, 2019). Despite being considered non-coding RNAs, recent work has shown some circRNAs, once exported to the cytoplasm, can indeed be translated in a 5′-cap-independent manner (Pamudurti et al., 2017; Yang et al., 2017). While nuclear export mechanisms of mRNA are well-characterized, little is known about how circRNAs are exported from the nucleus to the cytoplasm as they lack many of the common signals defining the export pathway used for export of mRNAs and other RNA species. Recent research suggests that the length of mature circRNA is measured in both human and *Drosophila* cells and may play an important role (Huang C. et al., 2018; Li Z. et al., 2019). It was shown that human DExH/D-box helicase UAP56 (DDX39B) is required for efficient nuclear export of long circRNAs (>1200-nt), whereas URH49 (DDX39A) controls the localization of short circRNAs (<400-nt). Similar length-dependent export was observed in *Drosophila* and was mediated by Hel25E, a homolog to DDX39A and B, demonstrating interspecies conservation of this mechanism.

The deposition of nuclear factors to pre-mRNA is necessary for nucleo-cytoplasmic transport and subsequent commitment to decay, storage or efficient translation to protein (Le Hir et al., 2001; Singh et al., 2020). circRNAs that are derived from introns (circular intronic RNAs, ciRNAs), remain in the nucleus and may regulate transcription of parental genes (Zhang et al., 2013; Zang et al., 2020) (Figure 1). Those derived from exons undergo nucleocytoplasmic transport and post-transcriptionally regulate gene expression (Figure 2).

circRNA serve as microRNA (miR) decoys and harbor copies of miR response elements (MREs). Unlike mRNA, circRNAs bound to miR are completely resistant to miR-mediated target destabilization (Hansen et al., 2013). One miR may have hundreds of binding sites in mRNA- and circRNA-targets and the interplay between the circRNA, miR and target mRNA generates competing endogenous (ce) RNA crosstalk. Connecting the patterns of ceRNA crosstalk draws the ceRNA regulatory network (Lan et al., 2019). For a comprehensive review on circRNA-miR interactions, please refer to Chandra (2018).

Mounting evidence posits circRNA and miR function independently to significantly regulate posttranscriptional gene expression. The circRNA CDR1as (antisense to the cerebellar degeneration-related protein 1 transcript, also termed as ciRS-7), contains 70 MRE, and ciRS-7 binding does not activate miR-mediated destabilization (Hansen et al., 2013). The expression of CDR1as is upregulated by the tissue-specific transcription factor *MyoD*, which has a critical role in muscle differentiation. By sequestering miR-7 and Argonaute, ciRS-7 was shown to derepresss miR-7 target mRNAs and activate embryonic muscle development in goat, e.g., insulin like growth factor 1 receptor (Li L. et al., 2019).

circRNAs also serve as protein decoys to regulate biological processes (Figure 2). For example, circ-Foxo3 can repress cell cycle progression by binding to G1 to S phase transition-related *CDK2* and *p21* (Zhang Z. et al., 2018). Also, circ-Foxo3 can retain the stress-associated proteins FAK and HIF1a in the cytoplasm to block their nuclear function and promote senescence of myocardial cells (Zang et al., 2020). The activity of circRNA ZNF609 in mouse and human myoblasts was shown to require nuclear protein binding and loading to polyribosomes (Legnini et al., 2017). For more on the association of circRNA with RBP and translation, please refer to Zang et al. (2020).

Emerging evidence indicates circRNAs are important during major biological processes and disease states, such as growth and development (Song et al., 2019); differential expression; neurodegenerative disease (Wang et al., 2018; Dube et al., 2019); malignancy (Li et al., 2015; Zhang Z. et al., 2018; Dube et al., 2019; Song et al., 2019). circRNA molecules are abundant in blood and saliva and are present in exosomes – secreted vesicles that serve as extracellular transport vehicles in the circulation (Figure 2) (Memczak et al., 2015). The confirmed prevalence and stability of circRNAs in body fluids and their spatial-temporal distribution indicates potential utility as diagnostic and prognostic biomarkers for diseases (Li et al., 2015).

Despite distinct patterns of tissue-specific expression (more below), some circRNA exhibit no difference in the abundance in plasma or serum between patients and healthy controls (Zhang Z. et al., 2018). More research is necessary to elucidate functions of circRNA before they find their way in clinical applied science. But, emerging evidence posits translational value of circRNAs to veterinary and biomedical sciences.

## Contemporary Methodology for Detection of, and Obstacles to, Characterizing circRNA

### Experimental Methods

The expression of circRNAs was an almost completely uncharacterized component of eukaryotic gene expression until sample preparation protocols and deep sequencing were advanced (Lasda and Parker, 2019). Whereas enrichment of mRNAs by selection of poly-adenosine (polyA) RNA species has been a default step in the process of library preparation, this step depletes circRNAs (Barrett and Salzman, 2016). Primer selection is another common variable in RNA-seq protocols. The most commonly used primer, oligo deoxythymine (dT) hybridizes to the polyA residues concentrated at the 3′ terminus of most eukaryotic mRNAs. Since circRNAs lack the 3′ polyA tail, use of oligo (dT) primer imposes bias against detection of circRNA. Instead, random hexamer primers and paired-end sequencing reactions are prudent approaches to detect circRNAs in total RNA preparations (Panda and Gorospe, 2018).

Protocols to enrich circRNAs in RNA samples have been established. In transcriptome analysis, depletion of rRNA is desirable since 90% of cellular RNA preparations are ribosomal RNA and only 5% is non-coding RNA or mRNA, respectively (Salzman et al., 2012, 2013; Jeck and Sharpless, 2014; Barrett and Salzman, 2016). Following rRNA depletion, circRNAs are not depleted. The use of RNA exonucleases to digest linear RNA isoforms is now widely used for circRNA enrichment from a total RNA preparation (Du et al., 2017; Verduci et al., 2019). Digestion of mRNA samples with RNAse R, a processive exonuclease that digests linear RNA, has been widely adopted to deplete samples of lncRNA, mRNA and other linear RNA species prior to library preparation, a technique also known as CircleSeq (Jeck et al., 2013). In 34 paired samples of only rRNA being depleted (rRNA-) and a combination of rRNA depleted with RNAse R treatment (rRNA−/RNAseR+), most of circRNAs (50–80% of highly expressed circRNAs, respectively) detected in the rRNA- dataset were validated in the rRNA−/RNAseR+ dataset as well (Zhang et al., 2020). This shows that while CircleSeq technique certainly enriches circular species, it is not a mandatory step for successful detection of circRNAs in the sample. However, limitations of both approaches have been recognized. Zhang et al. (2020) evaluated RNA-seq datasets from four species (human, fly, worm, mouse) and observed high variance in the efficiency of rRNA sequence depletion. This discrepancy was attributed to the limited species specificity of current RiboMinus transcriptome isolation kits available. Caveats in the RNA sample processing significantly alter circRNA quantification and quality controls are critical to measure reproducibility within replicate sample analysis and between studies. CircleSeq, for instance, has the capacity to generate deep coverage of circular and lariat products, but this approach can introduce an unintentional bias. The digestion step enriches circRNA species that are RNAse R-resistant. Drawbacks to the use of RNAse R are that a single nicking event unleashes exonuclease sensitivity, and the possibility of contaminating RNA endonucleases (Jeck and Sharpless, 2014).

circRNA isolation methodology with high confidence standards was described by Panda et al. (2017). While RNAse R digests linear transcripts, activity is limited on fragmented linear RNA and highly structured species such as transfer RNA and small nuclear RNA and small non-coding RNA. Therefore, an innovative method termed RPAD (RNase R treatment followed by Polyadenylation and polyA+ RNA Depletion) prepares circRNA of the highest purity by a two-step elimination of linear transcripts in the sample. Briefly, the procedure is to treat the total RNA sample with RNAse R to digest linear transcripts, followed by polyadenylate polymerase, which labels undepleted linear molecules bearing free 3′-OH ends. Last is incubation with oligo-dT to deplete residual polyA-containing RNAs from the circRNA preparation.

As a group, circRNA generally are less abundant than their linear RNA counterparts. However, with the advancement of RNA library preparation, the reliability of RNA-seq technology and new computational strategies, results of quantitative investigations reveal certain human and chicken circular transcripts are more abundant than their homologous mRNAs (Salzman et al., 2012; Ji et al., 2019).

### Bioinformatic Tools

Numerous algorithms have been developed to detect junction-spanning sequences in deep-sequencing data set (Table 1). Two approaches discover a back-splicing junction site specific for circRNAs: the pseudo-reference approach and the segmented-based computational analysis of non-linear exons and introns. All pipelines specify an external aligner, such as Bowtie or HiSat2. The aligner begins by filtering out reads that contiguously align to the genome and/or to the transcriptome (Gao and Zhao, 2018). Subsequent processing of the unaligned reads identifies those that align to a back-splice junction, which is identified by the algorithm employing either pseudo-reference or a split-alignment base. Multiple algorithm specific filters, including read counts, multiple sample detection and statistical scores, being the most common, are applied to limit the rate of false positive reads.

**TABLE 1:** Overview of the most common algorithms used to detect circular RNAs from RNA sequencing data.

Software	Mapper	Approach	Link and references
**Pseudo-reference algorithms**			
KNIFE	Bowtie Bowtie 2	Quantifies splicing events at both annotated and unannotated exon boundaries	Szabo et al., 2015
NCLscan	BWA Novoalign	Detects non-colinear transcripts: circRNA; *trans*-splicing events; fusion transcripts	Chuang et al., 2016
**Segmented-based algorithms**			
MapSplice	Bowtie	Finds splice junctions using approximate sequence similarity and without dependence on features or locations of the splice sites	Wang et al., 2010
Segemehl	*Per se*	Identifies fusion reads by implementing a matching strategy based on enhanced suffix arrays	Hoffmann et al., 2014
find_circ	Bowtie2	Performs *de novo* detection of back-splice junction	Venø et al., 2015
circRNA_finder	STAR	Predicts circRNA that are within 100 bases of splice sites	Hansen et al., 2016
CIRI	BWA-MEM	Uses maximum likelihood estimate to detect back-splice junctions using multiple-seed matching	Zhang et al., 2016
CircPRO	BWA-MEM, Bowtie2	Identifies circRNA with potential to be protein-coding or non-coding circRNAs	Meng et al., 2017
CIRC explorer2	TopHat/STAR	An annotating tool that parses mapping information from other aligners	Zhang F. et al., 2018

The first group of programs is a candidate-based approach (pseudo-reference), where a circRNA reference set of all possible combinations of scrambled exon–exon junctions is built from a gene annotation repository. The back-splice junction reads are aligned contiguously to the computational reference set by algorithms such as KNIFE and NCLscan (Li and Han, 2019) (Table 1). The efficiency of these algorithms to identify novel back-splice junctions is lower than algorithms employing the fragmented-based criteria.

The second group of programs, fragmented-based, is also referred to as segment- or split-alignment-based and relies on the identification of back-splicing junctions from the mapping information provided by aligning reads to the reference genome or transcriptome (Gao and Zhao, 2018; Li and Han, 2019) (Table 1). This group of programs has been used more frequently and the results provided have been comparable between different algorithms. CIRI, find_circ, circExplorer, circRNAseq and MapSplice have reproducibly identified circRNAs using reference exonic, intronic and intergenic sequences that have been deposited in specialized databases: circBase and CIRCpedia, among others (Aghaee-Bakhtiari, 2018) (Table 2). These algorithms provide versatility to input either single-end or paired-end sequencing data; the latter is considered to enhance the sensitivity and reliability of the data analysis. Consensus is needed to establish widely utilized quality control standards that measure the accuracy of circRNA expression levels to improve the validity of data comparison between studies (Szabo et al., 2017). The computational processing of circRNAs from populations of RNA sequences presents an opportunity to apply machine learning methods to large datasets.

**TABLE 2:** Overview of publicly available circular RNA databases.

Name/Species	Description	Link and references
**Databases containing solely human circular RNAs**		
Circ2Traits	Categorizes circRNA by potential involvement in diseases and potential interaction with disease related miRs	Ghosal et al., 2013
CircInteractome	Predicts and maps binding sites for RNA binding proteins and miRs on reported circRNAs	Dudekula et al., 2016
CircNet	First database to collect tissue-specific circRNA profiles and proposed circRNA-miR regulatory networks	Liu et al., 2016
circRNADb	Collects annotated protein-coding human circRNAs	Chen et al., 2016
CSCD	Collects cancer-related circRNAs	Xia et al., 2018
**Databases containing circRNAs from multiple species**		
StarBase/human, mouse, worm	Decodes various interaction networks, a component of starBase	Li et al., 2014
circBase/human, mouse, fly, worm, fish	Compiles publicly available circRNA datasets and the python scripts to process RNA seq data for discovery of circRNAs	Glažar et al., 2014
CIRCpedia_v2/human, mouse, rat, zebrafish, fly, worm	Allows users to search circRNAs with expression characteristics in various physiological and diseased tissues and cell types and provides conservation analysis of circRNAs between humans and mice	Dong et al., 2018
CircAtlas/human, monkey, mouse	Collects expression patterns, genomic features, functional annotations and conservation of circ RNAs derived from 44 normal tissues	Ji et al., 2019
CircFunBase/human, cattle, chicken, fly, monkey, mouse, pig, rat, rabbit, plants	Visualizes circRNA-miR interaction networks and the genome context of circRNAs	Meng et al., 2019
**Database containing plant circular RNAs**		
PlantcircBase	Collects circRNAs from plant RNA seq data and predicts miR-mRNA networks	Chu et al., 2017

Once detected by the algorithm, circRNAs in published data are deposited into one or more databases (Table 2). Some databases catalog results based on species of origin, tissue location and disease-association, whereas others focus in-depth on an assigned function, protein coding capacities and the scope of competing endogenous networks (Table 2). Questions we pose are: What is the specific threshold or the criteria for candidate circRNA results to be published in sanctioned databases? What are the research community standards for rigor and reproducibility and for the assignment of Digital Object Identifiers to ensure data remain discoverable, freely reusable and citable (Martone, 2014).

Computational reproducibility has become an integral issue in scientific research, particularly due to the rapid advancement of computer environments (Boettiger, 2015). Several initiatives aim to enhance science data reproducibility and data literacy, e.g., The Carpentries, Docker, and Snake Make. Data Carpentry provides hands-on workshops to equip scientists for successful big-data analysis, e.g., scripting and data management (Pugachev, 2019). Snake Make is a Python-based framework for formalizing data analysis that provides rules-guided workflow and portable execution environment (Köster and Rahmann, 2012). Docker Container shares code and dependencies necessary for smooth reproducibility of datasets made available with scientific publications (Boettiger, 2015). These tools are useful beyond the scope of circRNAs into all aspects of Omics and other research requiring extensive computational processing.

## Circular RNAs in Animal Health and Disease

Emerging data indicate circRNA connect feed-back loops in all life processes, from embryonic development to growth, differentiation and response to infection. Aberration of ceRNA networks is consequential to infectious disease, neoplastic transformation, developmental and degenerative disease. In the following sections, perspectives coming from circRNA investigations in livestock, companion animals and wild species are discussed and considered in relation to food production and biomedical science. We emphasize emerging issues and promising avenues to utilize circRNA to improve health of people and non-rodent animal species.

### Production Animals – Animal Protein Sourced From Meat, Eggs, and Dairy

Stunning advances have been made in the genetics of food-producing animals. Selection for certain production traits has created a very different livestock compared to the historical primitive breeds. For decades, breeding efforts have been undertaken to select for higher milk yield in dairy cows, faster muscle growth in cattle or larger eggs in poultry. With that in mind, genomic information alone explains only a part of the phenotypic variance in traits (Ibeagha-Awemu and Zhao, 2015).

Non-coding RNAs serve epigenetic mechanisms, in addition to DNA methylation, histone tail modification and chromatin remodeling, that have the ability to modify phenotype without altering chromosomal DNA (Waddington, 2012). Epigenetic modifications can be altered by external (climate, pathogens, nutrition) or internal (hormonal cues) environmental factors and have the ability to change gene expression and promote emergence of specific phenotypes in individuals. Understanding the epigenetic determinants of animal diseases and their role in pathogenesis, control, treatment and eradication represents a major opportunity to apply epigenetic markers for further improvement of animal productivity (Waddington, 2012). Skeletal muscle growth and differentiation (Luo et al., 2013; Nie et al., 2015), milk production (Yang et al., 2018) and egg laying (Adetula et al., 2018) are precisely regulated by hormonal and developmental cues with circRNA prominently serving as a rheostat to balance mRNA expression with non-coding RNA regulation.

#### Poultry – Meat

The genome-wide identification and function analysis of circRNAs in chicken skeletal muscle were characterized over the stages of embryonic development (Ouyang et al., 2018b). Thirteen-thousand circRNAs were identified in total RNA preparations of leg muscles of six female Xinghua chickens during day 11 (E11) and 16 (E16) of embryonic development and the 1st day post hatch (P1). circRNA were most abundant on day E16 followed by P1 (Table 3). Analysis of differentially expressed circRNA (DEcircRNA) reveals that not only do the circRNA accumulate over time, emblematic of the aging process (Westholm et al., 2015), but parental genes of the DEcircRNA are involved in the development of muscle cell structure and differentiation and activity.

**TABLE 3:** Summary of variables in studies of circular RNAs in non-rodent animal models.

Tissue	Total RNA sample		Software	Condition/disease involvement	Year published
	rRNA-depleted	circRNA enriched			
***Gallus gallus* (Chicken)**					
Skeletal muscle	×	×	CIRI	Embryonic muscle development	Ouyang et al., 2018a, b
Skeletal muscle	×	×	N/A	Embryonic muscle development	Chen et al., 2019
Liver	×	×	find_circ	Avian leukosis virus pathogenesis	Zhang et al., 2017
Spleen	×		CIRI	Avian leukosis virus pathogenesis	Qiu et al., 2018
***Sus scrofa* (Pig)**					
Brain	×		find_circ	Embryonic brain development	Venø et al., 2015
Heart	×		find_circ	Postnatal development	Liang et al., 2017
Liver					
Spleen					
Lung					
Kidney					
Ovarium					
Testis					
Muscle					
Fat					
Skeletal muscle	×	×	CIRCexplorer2	Embryonic muscle development	Hong et al., 2019
Mammary gland	×		CIRCexplorer2	Impact of heat stress on milk production	Sun et al., 2020
***Bos taurus* (Cow)**					
Mammary gland	×		CIRI	Casein content in milk	Zhang et al., 2016
Skeletal muscle	×	×	N/A	Prenatal and postnatal muscle differentiation	Li et al., 2018
***Ovis aries* (Sheep)**					
Skeletal muscle	×	×	find_circ	Prenatal and postnatal muscle differentiation	Li C. et al., 2017
***Canis familiaris* (Dog)**					
Heart	×		find_circ	Rapid atrial pacing model of atrial fibrillation	Shangguan et al., 2018
***Oryctolagus cuniculus* (Rabbit)**					
Carotid artery	×		CIRC explorer2	Atherosclerosis	Zhang F. et al., 2018
***Macaca mulatta* (Rhesus macaque)**					
Skeletal muscle	×	×	CIRC explorer	*Aging*	Abdelmohsen et al., 2015

By screening the circRNA sequences for MRE content, two miRs associated with skeletal muscle development and differentiation of muscle cells were identified: miR-206 and miR-1a. The linear *RBFOX* mRNA is template for the translation of RBFOX-splicing factors that are essential for the maintenance of skeletal muscle mass and proteostasis (Singh et al., 2018). Two circRNA isoforms derived from the *RBFOX* mRNA were identified that have MRE for miR-206 and miR-1a. The sequestration of miR-206 and/or miR-1a by RBFOX2 circRNA has the potential to downregulate the RBFOX-splicing factors. This would tilt the dynamic balance between muscle protein synthesis and tightly controlled protein degradation that maintains muscle mass. Results also revealed exonic circSVIL derived from the supervilin gene involved in myogenesis to be the most abundant and differentially expressed circRNA in all three developmental stages in the Xinghua chicken.

In their second study, the authors focused on molecular and biochemical experiments to explore in depth properties of circSVIL (Ouyang et al., 2018a). It was found to harbor binding sites for miR-203, a miR targeting *c-JUN* and *MEF2C* genes involved in muscle differentiation and proliferation in myoblasts (Lu et al., 2017). Expression of circSVI*L* increased from E11 to E14, suggesting it is active in muscle differentiation during late stage embryonic development. The high expression levels during late embryo development correspond to previously documented spatial-temporal patterns of circRNAs in human, fly and pig (Salzman et al., 2013; Venø et al., 2015; Westholm et al., 2015).

A most recent study by Ouyang et al. (2018b) and Chen et al. (2019) focused on experimental validation of their previous RNA sequencing data, where they observed *HIPK3* gene produced eleven circular isoforms. The qRT-PCR analysis confirmed circHIPK3 expression level in E16 was significantly higher than at E11 and P1. This circRNA was shown to bind miR miR-30a-3p and inhibit myoblast proliferation by targeting the *MEF2C* gene required for maintaining the differentiated state of muscle cells. By contrast, circHIPK3 was shown to promote proliferation and differentiation of chicken myoblasts. These results reaffirm the hypothesis circRNAs competitively bind miRs, upregulating targeted linear mRNA. Interestingly, both miR-30a-3p and circHIPK3 followed the same expression trend – the levels decreased sharply in the first 2 days the cells were cultured in the differentiation media and then increased to a steady state in the differentiated cells. Further studies are warranted to map these ceRNA networks and document other miRs regulated through these circRNAs.

As the US is the world’s largest poultry meat producer and second largest poultry meat exporter, scientific knowledge of circRNAs regulating biological processes would be of practical value for US poultry producers nationwide. Given the significant progress that was made in the genetics of food-producing animals, more research into epigenetic activity and potential application of non-coding RNA technology has yet unrealized potential.

#### Swine – Meat

Similar to the observations in poultry (Ouyang et al., 2018a), an abundance of circRNA have been discovered in total RNA preparation from muscles of domestic pig (Liang et al., 2017). In skeletal muscle, circRNAs were differentially expressed in age matched controls between day 0, 30, and 240. Comparison of circRNA profiles at day 0 and day 30 implicated distinct temporal profiling related to postnatal growth and muscle development. CircRNAs generated from transcripts encoding proteins associated with glycosaminoglycan metabolism or regulation of calcium channels were differentially expressed in the day 30 to day 240 comparison. The most abundant circRNAs originated from transcripts related to muscle hypertrophy, including the *myosin* gene family.

While Liang et al. (2017) profiled circRNAs in the postnatal pork muscle tissue, Hong et al. (2019) analyzed circRNA expression profiles during embryonic skeletal muscle development in Duroc pigs. Their analysis used RNAse R-enriched samples harvested at 33, 65, and 90 days before birth (Hong et al., 2019). Significantly fewer circRNAs were found to be expressed on day 90 when compared to the other two time points, an interesting find considering the main muscle fiber development in pigs occur at 33 and 65 days post-coitus, indicating circRNA may have crucial functions in the initiation stage of skeletal muscle development. When exploring the parental sequences of the circRNA, regardless of the time point, they identified several abundant circular candidates derive from *myosin* gene family, similar to the observations made by Liang et al. (2017) in postnatal muscle tissues. They constructed circRNA-miR-mRNA networks between several key genes in myogenesis and circRNAs that revealed *PITX2* and *FGF2* are regulated by multiple circRNAs. These findings resonate with previous results that translation control of myosin RNA determines the quality of pork by regulating total fiber number, intramuscular fat content, water holding capacity and meat color in Berkshire pigs, a porcine breed known for its outstanding meat quality (Lim et al., 2015). The utility of circRNAs in regulating *myosin* gene expression warrants more in-depth investigations to develop technologies useful to the porcine meat industry. Another significant impact of the study of porcine embryonic muscle tissue was the identification of over 7,000 circRNAs, which at the time, was far more that the entire existing database of porcine circRNAs. This important advance points out the value of using RNAse R treatment prior to RNA sequencing to enrich circular species.

The authors of a more recent study decided to look into the circRNA profiles in mammary gland of lactating sows under heat stress (Sun et al., 2020), since it was reported that circRNAs are expressed in bovine mammary glands and could regulate milk content (Zhang et al., 2016). Even though swine milk is not a primary product for human consumption, quality and yield of milk in lactating sows plays an important role for feeding viable offspring raised for meat. Several previous reports suggested a potential direct correlation between environmental temperature and milk yield (Black et al., 1993; West, 2003; Liu J. et al., 2019). Heat-stressed lactating sows reduce their feed intake and their declined milk production does negatively affect piglet growth and development during lactation. Thermal stress is a common occurrence in high-yielding swine and identifying approaches to manage milk production is important for the sustainable and profitable porcine industry.

RNA sequencing analysis by Sun et al. (2020) revealed heat stress also significantly decreased the levels of casein family genes, *CSN1S1, CSN1S2, CSN3* in swine. The analysis of circRNA transcriptomes between non-heat-stressed and heat-stressed sows revealed 50 DE circRNAs between the groups. By performing Pearson correlation analysis between DE circRNAs and DE mRNAs, authors identified significant interactions between DE circRNAs and four lactation-related coding genes (*CSN1S1, CSN1S2, CSN3, WAP*) that were annotated by GO enrichment analyses. One circRNA, circCSN1S1_2, specifically stood out and was positively associated with the expression of the *CSN1S1*, *CSN1S2*, *CSN3*, and *WAP* genes. Furthermore, the MRE identified in circCSN1S1_2 were predicted to competitively bind miR-204 to increase expression of parental gene, *CSN1S1*. These trends were similar to observations in dairy cows (more below) (Zhang et al., 2016).

#### Poultry – Eggs

Consistent egg production is a characteristic of healthy hens and depends on hormonal and developmental regulation. Infection is the major concern to avoid in the production of high quality and quantity of poultry meat and eggs. Avian leukosis virus (ALV) infection heightens mortality of hens, reduces egg laying performance and diminishes the quality of eggs by reducing size and increasing fragility of the shell (Zhang et al., 2017; Qiu et al., 2018). The retrovirus infection is a major pathogen of both broilers and layer-type chickens and leads to enormous economic losses in the developing world and global poultry industry (Zhang et al., 2017).

ALV strain J (ALV-J) was first isolated from commercial broilers in 1988 in the United Kingdom and has since spread to other countries (Payne et al., 1991). ALV-J was found to be associated with myeloid leukosis in meat-type and layer-type chickens (Payne et al., 1991; Fadly and Smith, 1999; Cheng et al., 2010). As a result of strict eradication programs, ALV-J reportedly has been eliminated from breeding flocks, but ALV still remains an issue in broiler flocks (Li et al., 2016). Even though the myeloid neoplasm progresses slowly in commercial poultry, the virus infection delays growth and enhances susceptibility to secondary infections. Considering that there is no vaccine for ALV yet, there is strong incentive to identify efficient strategies to promote resistance to ALV-J-induced tumor formation and to breed chickens naturally resistant to this pathogen. The potential to use circRNA as predictors of resistance to the viral infection or as biomarkers of ALV pathogenesis has been posited in recent studies.

Chickens vary in susceptibility or resistance to ALV infection through activity of divergent receptors (Plachý et al., 2017) and cell-endogenous miRs (Li et al., 2012). Four differentially expressed miRs with MREs in nearly 500 mRNAs were found significantly enriched in liver tumor tissues extracted from ALV-J positive chickens (Wang et al., 2013). The predicted target genes were found to be involved in tumorigenesis-related pathways, MAPK signaling pathway and Wnt signaling pathway. Zhang et al. (2017) postulated circRNAs are protective against ALV-J infection and tumorigenesis in chickens. To evaluate endogenous circRNAs as initiators of protective immune effects, circRNA sequencing on total RNA preparations was performed.

circRNA sequences were obtained from three liver tissue samples of ALV-J resistant chicken and three liver tissues of ALV-J susceptible chicken. The results identified 1,800 circRNAs. Thirty-two circRNA were differentially expressed, with 12 upregulated in ALV-J resistant chickens. Gene ontology identified the functionality of the parental transcripts in upregulated circRNAs to be immune-related pathways, such as antigen receptor signaling and B cell activation, which suggested involvement of the circRNA in antiviral immunity. Also, some of the predicted miR target genes, such as *eIF4E* and *PI3K*, are known to positively regulate the mTOR pathway, thus activating protein synthesis. mTOR activity is essential for cell growth and its dysregulation is linked to uncontrolled cell proliferation in cancer. Therefore, circRNA attenuation of mTOR activity by sequestering miRs was posited to be tumor-suppressive (Zhang et al., 2017).

Qiu et al. (2018) sequenced total RNA isolated from spleen of 20-weeks-old ALV-positive and ALV-negative chicken characterized as black-bone silky fowls. They reported the apparent loss of approximately 30% of circRNAs in ALV-infected chickens, aligning with the reported downregulation of circRNA in response to viral pathogens (Li X. et al., 2017; Liu C. X. et al., 2019). They observed correlation between genes deriving from differentially expressed circRNA, the parental mRNAs and complementary miRs, and the generalized upregulation of immune-related genes. By constructing ceRNA networks, authors speculated circRNAs contribute to ALV-mediated tumorigenesis. While the relationship between circRNAs and ALV pathogenesis has been based on computational predictions that remain to be validated experimentally, circRNAs have also been postulated to mediate bacterial and viral diseases in humans (Haque and Harries, 2017; Huang et al., 2017; Tagawa et al., 2018). Taken together, these findings provide inferences circRNAs regulate disease initiation and progression in poultry.

#### Cattle

Total RNA was isolated from rear quarters of the mammary glands of four lactating Holstein cows at a dairy farm during the lactation cycle at 90- and 250-days post-partum (Zhang et al., 2016). Three-thousand genes in the mammary gland were predicted to produce circular transcripts and many were related to genes encoding components of vesicles, endoplasmic reticulum, and mitochondrial lumen that contribute to milk protein biosynthesis and secretion. Four of the four casein-encoding genes (*CSN1S1*, *CSN1S2*, *CSN2*, and *CSN3)* were found to express circRNAs in the mammary gland. Expression levels were up to nine-fold increased on day 90 compared to day 250, correlating with the observed decrease in milk casein production as lactation wanes. Furthermore, cattle casein circRNAs had an abundance of MRE for miR predicted to target CSN1S1 and CSN2 mRNAs. The results support the hypothesis CSN circRNA regulate milk production through competitive binding of miR that upregulates translation of Casein proteins (Figure 2).

circRNAs also play a role in cattle muscle development. To identify circRNAs in skeletal muscle, three longissimus muscle samples were obtained from Qinchuan cattle at embryonic day 90 and adulthood at 24 months (Li et al., 2018). They identified circFGFR4 is a highly expressed circRNA with binding sites for miR-107 that decreased expression of established myogenic markers, *MyoD* and *myogenin* and the formation of myotubes. The validation experiments established circFGFR4 binds miR-107, positively influencing myoblast differentiation and development. Previously, abundant miR107 was identified in cattle skeletal muscle and shown to progressively decrease in quantity between embryonic and adult stage (Sun et al., 2013). It is possible increased expression and/or availability of circFGFR4 sequesters miR107 progressively.

Another report on circRNA in cattle skeletal muscle tissue profiled prenatal and postnatal *longissimus dorsi* muscle in sheep (Li C. et al., 2017). This study relied on computational prediction models to construct competing endogenous RNA networks to be tested in future validation studies. Nonetheless, the authors posited circRNAs have multiple target sites for miRs previously associated with muscle development, e.g. miR143, miR-133 and miR-23. Further analysis of mRNA targets and KEGG pathway analysis revealed the circRNA derived from parental genes in muscle growth and development signaling pathways. More data acquisition will be important to grasp the role of circRNA in sheep.

### Animal Models for Translational Research

Whether naturally-occurring or experimentally-induced, study of circRNA in farm animals contributes richly to the fruits of biomedical research. Animals and people suffer from breast cancer, melanoma, obesity and even psychological disorders, like anxiety and depression. Comparative basic science investigation of pathogenesis and molecular genetics, as well as risk factor assessment have been invaluable resources to learn how to cure disease of animals and people (Morello et al., 2011; Makielski et al., 2019).

#### Swine

The biology of the brain is prominently regulated by non-coding RNAs (Nie et al., 2019). In the mammalian brain, circRNAs contribute to neurological development and neuronal tissue differentiation (Jeck et al., 2013; Rybak-Wolf et al., 2014; You et al., 2015; Venø et al., 2015; Zhao et al., 2016; Floris et al., 2017). There is strong bias for circRNA upregulation in neural tissues of adult mice that has suggested the disruption of ceRNA networks contributes to aging (Gruner et al., 2016). Indeed, there is significant association between the expression of certain circRNAs like circHOMER1 in the brain and Alzheimer’s traits (*p* = 10^–12^) (Zhao et al., 2016; Huang J. L. et al., 2018).

The stages of fetal brain development has been well-characterized in swine and Venø et al. (2015) used the swine model to profile developmentally regulated circRNAs. The expression of *Sus scrofa* circRNAs was measured in the cortex, hippocampus, cerebellum, brainstem and basal ganglia at several time-points from early embryonic development to the time of birth. The authors reported clear fluctuations in circRNA expression as the gestation progressed. But, in contrast to results in poultry (Ouyang et al., 2018a), porcine circRNAs increased as the gestation progressed, peaking at embryonic day E60, followed by a steep decline between E80 until birth. The spatial-temporal expression profile correlated with differentiation of the fetal cortex, specifically, neuronal migration of E60 brain to acquire the gyri and sulci characteristic of the gyrencephalic brain.

Remarkably, porcine circRNAs represented over 10% of all expressed genes (range 6 – 14%) (Venø et al., 2015). The introns flanking circularized exons frequently have complementary SINEs, suggesting these RNA motifs drive circRNA by facilitating base pairing between the repetitive sequence of flanking introns, similar to viroid RNA motifs that were discussed above. This study constitutes the first circRNA profiling of the brain development of a large animal (Table 3).

Liang et al. (2017) used the Guizhou miniature pig model to perform spatial-temporal profiling of nine different organs (heart, liver, spleen, lung, kidney, ovarium, testis, muscle, and fat) and skeletal muscle tissues. Sampling was performed at three post-natal developmental stages, day 0, 30, and 240 after birth. They identified dynamic expression changes and the high abundance of circRNAs in the testicular tissue. Notably, Sry circRNA, previously reported to be predominant over linear *Sry* transcript in mouse testis (Capel et al., 1993), was not found. Significant new findings were the identification of several porcine heart-specific circRNAs related to hypertrophic cardiomyopathy, recapitulating circRNAs in human heart development and pathogenesis of cardiac diseases (Sonnenschein et al., 2019). Results identified MREs in 30% of the porcine circRNAs, implicating functionalities beyond miR sponging for porcine circRNAs. Similar to other non-coding RNAs, circRNA have the potential to modulate the local free concentration of RBPs by direct binding and by occupying their binding sites in target RNAs. Given circRNA are abundant, stable and only recently appreciated, their activity may profoundly balance gene expression and response to metabolic stress and infection.

Approximately 90% of the porcine circRNA splicing regions aligned with the mouse genome and one-fourth of the splice sites were identical between these two species, showing significant conservation between the profiles of porcine and murine circRNAs (Venø et al., 2015). CircRNA of Guizhou miniature pig originated from orthologous loci identified in murine (25%) and human (87%) circRNA profiles, confirming circRNA sequence conservation is prevalent between these species (Liang et al., 2017). In addition, authors report almost half of the detected porcine circRNAs were flanked by long introns, matching the prior observations made in humans (Zhao et al., 2019). Perhaps an outlier, chicken exon-intron circHRH4 was also flanked by long partial introns (Qiu et al., 2018). These observations could support a theory that repetitive sequences in flanking introns and RBPs are brought together to facilitate back-splicing (Figure 2). However, bovine circRNAs appear to lack repetitive sequences in flanking introns to mediate circularization (Zhang et al., 2016). Future studies are warranted to define the intrinsic determinants of RNA circularization that are conserved among different clades of animals.

#### Companion Animals

Even though companion animals with a defined pedigree are a promising translational model (Nichols et al., 2009; Pinho et al., 2012; Hytönen and Lohi, 2016; Supsavhad et al., 2016; Hicks et al., 2017), to our knowledge only two studies have profiled circRNA expression (Table 3). Both studies investigated circRNAs in the context of cardiovascular diseases (Shangguan et al., 2018; Zhang F. et al., 2018).

Chronic rapid atrial pacing (RAP) in dogs is an established model to study atrial fibrillation (AF), a common arrhythmia, which in humans leads to heart failure (Kijtawornrat et al., 2008). RNA sequencing of atrial tissues from dogs that suffered RAP and healthy controls showed differences in the expression of circRNAs (Shangguan et al., 2018). Gene Ontogeny identified transcripts encoding cytoskeleton and ion channel activity that participate in the process of atrial structural and electronic remodeling. An extensive network of differentially expressed circRNAs and AF-related miR were associated with the development of AF.

The relationship between circRNAs and atherosclerosis was investigated after inducing atherosclerotic plaques on the right carotid arteries in New Zealand white male rabbits by endothelial injury and high fat diet (Zhang F. et al., 2018). Differential analysis of circRNA, miR and mRNA revealed significant differences between the treatment and control group that mapped to immune response, cell adhesion, T-cell activation and cytokine production pathways. The study found hundreds of circRNAs and mRNAs having similar MRE, setting up potential widespread competition for miRs in each subnetwork. Consistent with a role for circRNA, study of human circANRIL identified changes affect human smooth muscle vascular tissue preventing the development of changes leading atherosclerosis (Kohlmaier et al., 2016; Xu et al., 2019). Further studies are warranted to define whether generalized dysregulation of circRNA predisposes to atherosclerosis.

#### Non-human Primates

Numerous experiments have established that muscle in humans is one of the tissues that is enriched in circRNAs. The dystrophy gene is among the first genes identified to generate circRNAs in skeletal muscle (Surono, 1999; Zhang et al., 2019). It is thought that one of the proposed models for circRNA biogenesis, exon skipping, may provide a cure to Duchenne muscular dystrophy patients by generating circular isoforms that skip over a region in the gene that accounts for 60% of the cases exhibiting this disease phenotype (Zhang et al., 2019). As mentioned above, circANRIL in INK4 locus was shown to protect from atherosclerosis by preventing oxidative stress and proliferation of smooth muscle cells in the endothelial vascular wall, and inducing apoptosis by activating p53 (Holdt et al., 2016; Shi et al., 2020). The circ-ZNF609, which was found to act as miR sponge to promote myoblast differentiation, was the first reported protein coding circRNA in skeletal muscle (Zhang et al., 2019). For a comprehensive review on circRNAs as regulators of myogenesis, please refer to Zhang et al. (2019).

On the other hand, Abdelmohsen et al. (2015) profiled expression of circRNAs in a non-human primate muscle tissue. CircRNAs were computationally predicted in total RNA isolated from 24 samples of the quadriceps leg muscle (*vastus lateralis*) from young, middle-aged and elderly rhesus macaques (age range 0.003–41 years old). The majority of the detected circRNAs were similar among the different age groups, but 19 were significantly downregulated circRNA as individuals aged. The significance of circRNA to muscle development, musculoskeletal pathology and age-related changes in primates is likely to be informed by studies of swine and poultry skeletal muscle with high statistical power.

#### Expression Landscape of circRNA: Man, Macaque, and Mouse

In a landmark publication, Ji et al. (2019) explored the landscape of the human, macaque, and mouse transcriptomes across 19 primary tissues, identifying 70,186 evolutionarily conserved circRNAs between these species and confirming the highest prevalence in neural and testicular tissues. Sixty-seven percent of the circRNA were detected in only one tissue. By constructing long-insert RNAseq libraries (400–800 base), in combination with long reads and high sequencing depth, endogenous circRNA sequences could be reconstructed, thereby increasing reliability of circRNA annotation. Notable was the classification of comparable tissue expression patterns between circRNA and RBP, implicating coordinate activity of circRNAs and RBPs in modulating the local concentration of linear RNAs or their cognate binding sites. The expression variance of the circRNA was low across different individuals, suggesting that circRNA expression is under tight regulation rather than being stochastic.

## Concluding Remarks

**FIGURE 3:**
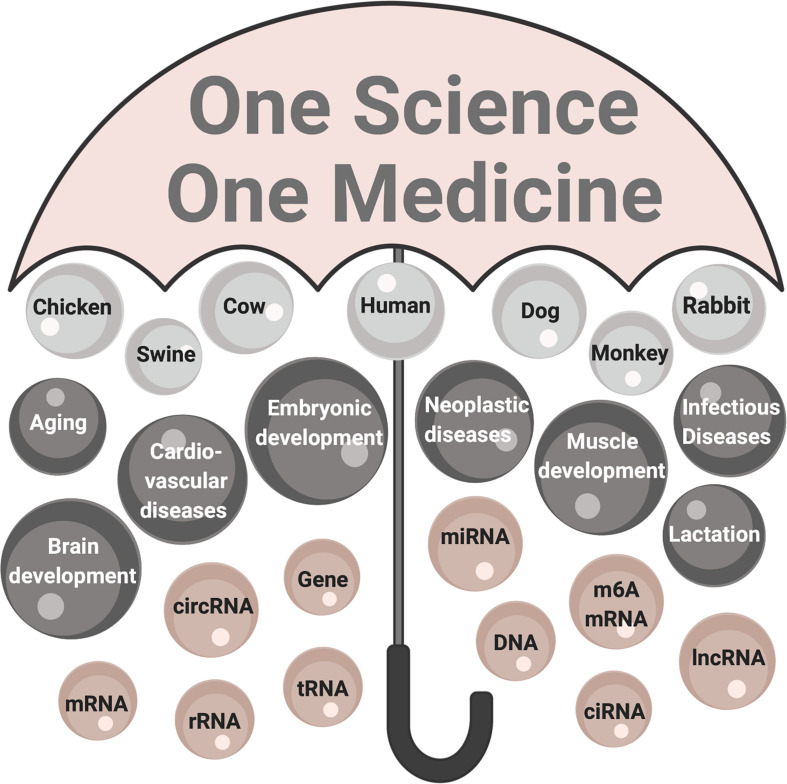
The One Health perspective of circRNA research. The umbrella labeled One Science One Medicine encompasses circRNA research in human and animal species that is contextual to healthy people, animals, environments. The significance of circRNA research: organisms are top-level components (light gray); processes and conditions affected are mid-level components (dark gray); and fundamental components are molecules (mauve). mRNA, messenger RNA; circRNA, circularized RNA; rRNA, ribosomal RNA; tRNA, transfer RNA; miR, microRNA, m^6^A mRNA, mRNA modified by adenine modification; ciRNA, circularized intronic RNA; lncRNA, long non-coding RNA.

One Health is a systems biology vision that encompasses the health of people, animals and global environment under one large umbrella (Zinsstag et al., 2011). Under the One Health umbrella, circRNA align innovative basic research that has elucidated new feed-back loops interconnecting processes and conditions common between people and animals (Figure 3). Even though the existence of circRNA has been known for nearly 50 years, circRNA literature has doubled in the past 5 years – largely through computational biology and RNAseq technology accompanied by molecular biology tools. circRNAs are now known to regulate biological processes beginning at embryonic development, throughout growth cycles and the aging process. Investment is warranted to elucidate competing endogenous RNA networks in animals and people and integrate the genetic and epigenetic expression patterns that once appeared stochastic to prevent disease. Implementing the One Health perspective over the next 5 years is to apply innovative basic research findings to the invention of diagnostic tools and therapeutic application of circRNAs in veterinary and human medicine.

## Author Contributions

KB-L outlined the manuscript, tables, and figures. DZ wrote the manuscript and created the figures. DZ and KB-L finalized the manuscript. Both authors contributed to the article and approved the submitted version.

## Conflict of Interest

The authors declare that the research was conducted in the absence of any commercial or financial relationships that could be construed as a potential conflict of interest.
